# Dietary total antioxidant capacity and the occurrence of metabolic syndrome and its components after a 3-year follow-up in adults: Tehran Lipid and Glucose Study

**DOI:** 10.1186/1743-7075-9-70

**Published:** 2012-07-31

**Authors:** Zahra Bahadoran, Mahdieh Golzarand, Parvin Mirmiran, Niloofar Shiva, Fereidoun Azizi

**Affiliations:** 1 Nutrition and Endocrine Research Center, Research Institute for Endocrine Sciences, Shahid Beheshti University of Medical Sciences, No 24 Parvaneh St., Yemen St., Chamran Exp., Tehran 19395-4763, Iran; 2 Department of Clinical Nutrition and Dietetics, Faculty of Nutrition Sciences and Food Technology, National Nutrition and Food Technology Research Institute, Shahid Beheshti University of Medical Sciences, No 46 Arghavan-e-gharbi St., Farahzadi Blv., Shahrak-e-Ghods, 19395-4741 Tehran, Iran; 3 Endocrine Research Center, Research Institute for Endocrine Sciences, Shahid Beheshti University of Medical Sciences, No 24 Parvaneh St., Yemen St., Chamran Exp., Tehran, 19395-4763 Iran

**Keywords:** Dietary total antioxidant capacity, Antioxidant-rich foods, Metabolic syndrome

## Abstract

**Background:**

There is growing evidence that dietary antioxidants could have favorable effects on the attenuation and prevention of metabolic disorders. In the current study we investigated the association of dietary total antioxidant capacity (TAC) and metabolic syndrome (MetS) components and the occurrence of the MetS during a 3-year follow-up.

**Methods:**

This longitudinal study was conducted in the framework of Tehran Lipid and Glucose Study, between 2006–2008 and 2009–2011, on 1983 adults, aged 19–70 y. The usual intakes of participant were measured using a validated semi-quantitative food frequency questionnaire and dietary TAC was estimated at baseline. The MetS components were assessed at baseline and 3 years later. Multiple logistic regression models were used to estimate the occurrence of the MetS and its components according to dietary TAC quartile categories.

**Results:**

The mean age of participants was 40.4 ± 13.0 y, and mean BMI was 27.03 ± 4.9 kg/m^2 ^at baseline. After adjustment for potential confounding variables, TAC was associated with MetS components at baseline. Participant with highest TAC score had lower weight and abdominal fat gain during the 3 year follow-up. The chance of having the MetS, abdominal obesity and hypertension after 3 years decreased across the increasing dietary TAC quartile (*P* for trend < 0.01). Dietary TAC more than 1080 μmolTE/100 g of food, resulted in a 38% decrease in the risk of central obesity (OR = 0.62, 95% CI = 0.38-0.99).

**Conclusion:**

We demonstrated that higher dietary antioxidant intakes have favorable effects on metabolic disorders and, more interestingly, prevent subsequent weight and abdominal fat gain during a 3-year follow-up.

## Background

Metabolic syndrome is a common multi-factorial disorder including abdominal obesity, insulin resistance, hypertension and dyslipidemia, leading to accelerated atherosclerosis and increased risk for diabetes [1,2]. There is growing evidences that this systemic disorder may be a result of oxidative stress [3,4]. It has been indicated that an impaired balance between free radical production and an impaired antioxidant defense system resulting in accumulation of oxidative damage, could playing important roles in pathological conditions such as insulin resistance, altered energy production and endothelial dysfunction as main risk factors of metabolic syndrome [5]. Dietary antioxidants have been reported to protect against oxidative damage and its complications [6] but the effects of antioxidants on the risk of MetS and related metabolic disorders have not been clarified and the results of clinical trials in this regard are inconsistent [7-9]. Since the evaluation of single antioxidant component may not reflect the total antioxidant power of diets and other possible interaction or synergetic effects of antioxidants, the concept of dietary total antioxidant capacity (TAC) was recently developed as a novel and relevant tool for assessment of the beneficial effects of dietary antioxidants [10,11]. The TAC of foods describes the ability of food antioxidants to scavenge free radicals and it is measured using the oxygen radical absorbance capacity (ORAC) assay [12]. Dietary TAC has been positively associated with plasma total antioxidant capacity [13] and has also related with higher diet quality, based on common indexes such as the Mediterranean diet and the Healthy Eating Scores [14]. Recently cross-sectional studies reported that dietary TAC was inversely related to plasma high-sensitive C reactive protein (hs-CRP) as a marker of systemic inflammation and mediator of metabolic disorders [15], central adiposity, oxidized LDL-C concentration [13], and diabetes biomarkers [16]. Food selection based on dietary TAC in a crossover intervention also modified antioxidant intakes, systemic inflammation and liver function [17]. Although some beneficial aspects of a high TAC diet on health promotion and disease prevention have been investigated, more longitudinal and cohort studies are required to confirm these results and be prioritized in public health strategies and dietary recommendations. In the present study we investigated whether dietary TAC could affect the occurrence of MetS and related metabolic disorders after 3-year follow-up in Tehranian adults.

## Experimental methods

### Study population

This study was conducted within the framework of the Tehran Lipid and Glucose Study (TLGS). Briefly, TLGS is a community-based prospective study conducted to investigate and prevent non-communicable diseases, in a representative sample of residents, aged ≥ 3y, from district 13 of Tehran, the capital city of Iran. The first phase of the TLGS began in March 1999 and data collection, at three-year intervals, is ongoing [18].

A total of 12 523 participants were examined at the third phase of the TLGS (2006–2008). Of these, 4920 participants were randomly selected for dietary assessment, categorized by age and sex categorization; data obtained using the food frequency questionnaire (FFQ) was available for 3462 participants at this phase [19]. For the current study, 2799 men and women aged 19–70 y, were recruited. Subjects were excluded if they under- or over-reported dietary intakes (less than 800 kcal/d or more than 4200 kcal/d, respectively), or they were on specific diets. The final sample at baseline (2006–2008), included 2567 adults (1129 men and 1438 women) and the mean duration of the follow-up was approximately 3 years. Of the 2567 initial participants who attended the baseline examination, 629 subjects who had no follow-up information on anthropometrics and biochemical measurements were excluded and final analysis of data was performed for 1938 (75.5%) participants.

Informed written consents were obtained from all participants and the study protocol was approved by the research council of the Research Institute for Endocrine Sciences, Shahid Beheshti University of Medical Sciences.

### Dietary assessment and dietary TAC calculation

Dietary data were collected using a validated semi-quantitative food frequency questionnaire (FFQ) with 168 food items. Trained dietitians, with at least 5 years of experience in the TLGS survey, asked participants to designate their intake frequency for each food item consumed during the past year on a daily, weekly, or monthly basis. Portion sizes of consumed foods reported in household measures were then converted to grams [19].

Because the Iranian Food Composition Table (FCT) is incomplete, and has limited data on nutrient content of raw foods and beverages, to analyze foods and beverages for their energy and nutrient content we used the US Department of Agriculture (USDA) FCT [17].

Dietary TAC was estimated based on the oxygen radical absorbance capacity of selected foods reported by Nutrient Data Laboratory of USDA, and expressed as μmol of Trolox Equivalents per 100 grams of foods (μmolTE/100 g) [20].

### Lifestyle, anthropometrics and clinical measurement

Trained interviewers collected information using a pretested questionnaire. Information on age (years), current smoking (yes/no), educational level (illiterate, primary, academic and advanced academic education) and physical activity (MET-h/wk) were assessed at baseline examination (2006–2008). Smoking status was obtained using face-to-face interviews and subjects who smoked daily or occasionally were considered current smokers, while non-smoker included those who had never smoked or those who had quit smoking. Physical activity level was assessed using the Krishna et al. questionnaire [21] to obtain frequency and time spent on light, moderate, hard and very hard intensity activities according to the list of common activities of daily life over the past year. Physical activity levels were expressed as metabolic equivalent hours per week (METs h/wk).

Anthropometric measurements were assessed at baseline and again after a 3-year follow-up by trained staff. Weight was measured to the nearest 100 g using digital scales, while the subjects were minimally clothed, without shoes. Height was measured to the nearest 0.5 cm, in a standing position without shoes, using a tape meter. Waist circumference (WC) were measured to the nearest 0.1 cm (at anatomical landmarks), at the widest portion, over light clothing, using a soft, tape meter, without any pressure to the body. Body mass index was calculated as weight (kg) divided by square of the height (m^2^).

For blood pressure (BP) measurements, after a 15-minute rest in the sitting position, two measurements of BP were taken, on the right arm, using a standardized mercury sphygmomanometer; the mean of the two measurements was considered as the participant's BP.

### Biochemical measurement

Fasting blood samples were taken after 12–14 h, from all study participants at baseline and after a 3-year follow-up. Fasting plasma glucose (FPG) was measured by the enzymatic colorimetric method using glucose oxidase. Triglyceride (TG) level was measured by enzymatic colorimetric analysis with glycerol phosphate oxidase. High-density lipoprotein cholesterol (HDL-c) was measured after precipitation of the apolipoprotein B containing lipoproteins with phosphotungstic acid. Analyses were performed using Pars Azmoon kits (Pars Azmoon Inc., Tehran, Iran) and a Selectra 2 auto-analyzer (Vital Scientific, Spankeren, Netherlands). Inter- and intra- assay coefficient of variation of all assays was < 5%.

### Definition of metabolic syndrome and its components

Cardio-metabolic risk factors for the metabolic syndrome were defined according to the diagnostic criteria proposed by NCEP ATP III [22], and new cutoff points of waist circumference for Iranian adults [23]; the syndrome was characterized as having at least 3 of the metabolic abnormalities: 1) Hyperglycemia as FPG ≥ 100 mg/dL (5.6 mmol/L) or drug treatment of impaired fasting glucose, 2) Hypertriglyceridemia as serum TG ≥ 150 mg/dL (1.69 mmol/L) or drug treatment, 3) Low HDL-c as serum HDL-cholesterol < 40 mg/dL (1.04 mmol/L) for men, and < 50 mg/dL (1.29 mmol/L) for women or drug treatment, 4) Hypertension as BP ≥ 130/85 mmHg or drug treatment for hypertension, and 5) Abdominal obesity as WC ≥ 95 cm for both genders.

### Statistical methods

All statistical analysis were conducted using SPSS (Version 16.0; Chicago, IL), and *P* values < 0.05 were considered significant. Participant characteristics, the mean of MetS components and the prevalence of MetS and its features were compared at baseline across quartile categories of dietary TAC, using the general linear models with adjusted for sex and age or the Chi-square test. Mean dietary intakes of participants were compared across quartile categories of dietary TAC using the general linear model with adjustment for sex, age (y, continuous), and energy intakes (kcal/d). The association of dietary TAC and MetS components at the first examination, in each quartile category, was measured by using the linear regression model with adjustment for sex, age (y, continuous), BMI (kg/m^2^, continuous), education (4 categories), smoking (yes or no), physical activity (MET-h/wk, continuous), total energy intake (kcal/d), percent of energy from dietary carbohydrate, protein, total fat, saturated fat, mono and poly-unsaturated fat, and total fiber (g/1000 kcal/d).

Changes of metabolic syndrome components, during the 3-year follow-up, were calculated as [(follow-up measure - baseline measure) / baseline measure] × 100, and were compared across TAC quartiles using the general linear models, adjusted for baseline confounding variables.

To estimate the odds ratio of MetS and components in each quartile category of dietary TAC at the second examination, participants with metabolic disorders at baseline were excluded from the analysis; based on this, the multivariable logistic regression models, adjusted for the above-mentioned potential confounders, analyses were conducted on 1447 subjects for the MetS, 1276 subjects for abdominal obesity, 1672 subjects for impaired fasting glucose, 1252 subjects for hypertriglyceridemia, 592 subjects for low-HDL-c, and 1577 subjects for hypertension. Dietary potassium intake (mg/1000 kcal/d) was additionally adjusted for the assessment of dietary TAC in relation to 3-year changes of systolic and diastolic blood pressure and the occurrence of hypertention.

To assess the overall trends of odds ratios of metabolic syndrome and its components across quartiles of dietary TAC, the median of each quartile was used as a continuous variable in the logistic regression models.

## Results

The mean age of participants was 40.4 ± 13.0 y, and mean BMI was 27.03 ± 4.9 kg/m^2 ^at baseline. Forty-seven percent of participants were men. The mean weight gain was 1.49 ± 5.06 kg (1.65 ± 5.3 kg in men and 1.34 ± 4.9 kg in women) during the 3-year period; lower weight gain during 3-year follow-up was observed in the participants with the higher TAC diet (0.9 kg in fourth quartile *vs.* 1.9 kg in first quartile, *P < 0.05).* The mean of dietary TAC was 962 ± 189 μmolTE per 100 g of food intakes (929 ± 186 in men and 986 ± 187 in women μmolTE/100 g). Participants in the highest TAC quartile category were more likely to be women than men (57 *vs.* 43%, *P* for trend <0.05), were older (37 *vs.* 43 years, *P* for trend <0.001), and were less likely to be current smokers (9.2 *vs.* 13.7%, *P* for trend <0.01). Participants in the highest TAC quartile also spent more time for leisure physical activity (*P* < 0.05). There was no significant difference between the educational status of participants across quartile categories (Table 1).

**Table 1:** Characteristics of participants by categories of dietary total antioxidant capacity at baseline: Tehran Lipid and Glucose Study¹

	**( *n* = 1938)**				
	** *Q1* **	** *Q2* **	** *Q3* **	** *Q4* **	** *P***^***2* **^
Total antioxidant capacity					
*Range*	< 842	842-958	959-1080	> 1080	
*Median*	764	906	1014	1161	
Age at baseline *(yr)*	37.0 ± 0.6	40.2 ± 0.5	41.4 ± 0.5	43.0 ± 0.5	*<0.001*
Men *(%)*	53.9	46.8	39.0	34.5	*<0.001*
Physical activity *(Met-h/week)*					
*Job activity*	29.2 ± 2.3	26.7 ± 2.3	22.5 ± 2.3	26.0 ± 2.3	*0.25*
*Leisure time activity*	8.2 ± 0.7	9.9 ± 0.7	10.2 ± 0.7	12.0 ± 0.7	*0.005*
*Total*	37.5 ± 2.5	36.6 ± 2.4	32.7 ± 2.4	38.1 ± 2.4	*0.41*
Current smoker *(%)*	13.7	11.0	10.8	9.2	*0.16*
Education status *(%)*					
*Illiterate*	2.5	1.7	2.3	2.7	*0.73*
*Primary education*	7.5	3.2	6.5	0	*0.81*
*Academic education*	81.1	90.3	83.9	91.3	*0.81*
*Advanced academic education*	11.3	6.5	9.7	8.7	*0.81*

^1^Data are age- and sex-adjusted mean ± SEM.

^2^*P for trend* across quartiles of dietary phytochemical index was calculated using Chi square test or linear regression models with adjustment for sex and age.

The mean ± SEM of the MetS components and the prevalence of MetS and its risk factors across categories of dietary TAC at baseline are presented in Table 2. There were no significant differences between the MetS and its components in the participants at baseline. The mean dietary intake of participants across dietary TAC quartile categories are shown in Table 3. There was no difference in total energy intakes and calorie intakes from macronutrients between the dietary TAC quartile categories but there was a significant decreasing trend in dietary energy density from lower to upper TAC scores. Dietary intakes of vitamin A, total carotenoids, vitamin E, C, and zinc were significantly higher in participants with higher TAC diet. Moreover, participants in the highest quartile of dietary TAC had consumed more whole grains, fruits, legumes, dairy and nuts.

**Table 2:** Metabolic syndrome components and prevalence of metabolic syndrome and its risk factors by categories of dietary total antioxidant capacity: Tehran Lipid and Glucose Study¹

	**( *n* = 1938)**				
	** *Q1* **	** *Q2* **	** *Q3* **	** *Q4* **	** *P***^***2* **^
Total antioxidant capacity					
*Range*	< 842	842-958	959-1080	> 1080	
*Median*	764	906	1014	1161	
Waist circumference *(cm)*	90 ± 0.5	89.0 ± 0.5	89 ± 0.5	89 ± 0.5	*0.44*
Fasting blood glucose *(mg/dl)*	93 ± 1.1	91 ± 1.1	91 ± 1.1	90 ± 1.1	*0.46*
Serum triglycerides *(mg/dl)*	148 ± 3.7	142 ± 3.7	143 ± 3.7	137 ± 3.7	*0.24*
HDL-c *(mg/dl)*	42.2 ± 0.4	41.8 ± 0.4	42.2 ± 0.4	42.5 ± 0.4	*0.71*
SBP *(mmHg)*	112 ± 0.6	112 ± 0.6	111 ± 0.6	111 ± 0.6	*0.75*
DBP *(mmHg)*	74 ± 0.4	74 ± 0.4	73 ± 0.4	73 ± 0.4	*0.12*
Abdominal obesity *(%)*	44.2	43.7	46.5	38.8	*0.10*
Hypertriglyceridemia *(%)*	34.5	31.8	30.7	31.1	*0.57*
Low HDL-c *(%)*	49.2	50.9	51.2	47.6	*0.65*
Hyperglycemia *(%)*	22.7	25.2	24.1	28.0	*0.28*
Hypertension *(%)*	12.6	17.4	14.5	15.5	*0.20*
Metabolic syndrome *(%)*	24.6	27.5	27.4	25.9	*0.69*

^1^ Data are age-adjusted mean ± SEM.

^2^*P for trend* across quartiles of dietary phytochemical index was calculated using Chi square test or linear regression models with adjustment for sex and age.

**Table 3:** Dietary intakes of participants by categories of dietary total antioxidant capacity: Tehran Lipid and Glucose Study¹

	**( *n* = 1938)**				
	** *Q1* **	** *Q2* **	** *Q3* **	** *Q4* **	** *P***^***1* **^
Dietary phytochemical index					
*Range*	< 842	842-958	959-1080	> 1080	
*Median*	764	906	1014	1161	
Energy intake *(kcal/d)*	2285 ± 29	2290 ± 29	2254 ± 29	2265 ± 29	*0.80*
Energy density*(kcal/d)*	107.9 ± 0.9	97.6 ± 0.9	91.3 ± 0.9	88.6 ± 0.9	*<0.001*
Carbohydrate *(% of total energy)*	58.1 ± 0.3	56.8 ± 0.3	57.2 ± 0.3	58.5 ± 0.3	*0.21*
Protein *(% of total energy)*	13.0 ± 0.1	13.7 ± 0.1	13.9 ± 0.1	13.9 ± 0.1	*0.32*
Fat *(% of total energy)*	30.5 ± *0.3*	31.8 ± 0.3	31.7 ± 0.3	31.1 ± 0.3	*0.09*
Saturated fatty acid *(% of total energy)*	9.7 ± 0.2	10.8 ± 0.2	10.6 ± 0.2	10.6 ± 0.2	*0.15*
Mono-unsaturated fatty acid *(% of total energy)*	10.8 ± 0.1	11.0 ± 0.1	10.9 ± 0.1	10.5 ± 0.1	*0.18*
Poly-unsaturated fatty acid *(% of total energy)*	6.7 ± 0.1	6.7 ± 0.1	6.5 ± 0.1	6.2 ± 0.1	*0.09*
Vitamin A *(mg/d)*	422 ± 13	493 ± 13	547 ± 13	557 ± 13	*<0.001*
Total carotenoids *(μg/d)*	8677 ± 278	9731 ± 274	10725 ± 274	10874 ± 277	*<0.001*
Vitamin E *(mg/d)*	11.3 ± 0.2	11.6 ± 0.2	11.6 ± 0.2	12.2 ± 0.2	*0.001*
Vitamin C *(mg/d)*	109 ± 3.5	133 ± 3.5	158 ± 3.5	185 ± 3.5	*<0.001*
Zinc *(mg/d)*	10.6 ± 0.1	11.3 ± 0.1	11.7 ± 0.1	11.7 ± 0.1	*<0.001*
Selenium *(μg/d)*	111 ± 1.4	112 ± 1.4	112 ± 1.4	108 ± 1.4	*0.10*
Whole grains *(g/d)*	65.0 ± 4.3	87.1 ± 4.2	106.3 ± 4.2	116.1 ± 4.3	*<0.001*
Fruits *(g/d)*	223 ± 10	303 ± 10	405 ± 10	566 ± 10	*<0.001*
Vegetables *(g/d)*	275 ± 8	300 ± 8	309 ± 8	298 ± 8	*0.028*
Legumes *(g/d)*	12.5 ± 1.0	16.7 ± 0.9	18.4 ± 0.9	21.7 ± 1.0	*<0.001*
dairy *(g/d)*	329 ± 11	447 ± 11	516 ± 11	596 ± 11	*<0.001*
Nuts *(g/d)*	5.1 ± 0.4	5.9 ± 0.4	7.7 ± 0.4	9.9 ± 0.4	*<0.001*

^1^ Data are age-and energy adjusted mean ± SEM.

^2^*P* values compared the dietary intakes of participants across quartiles of dietary phytochemical index using linear regression models with adjustment of sex, age and energy intakes.

Baseline associations of dietary TAC and the MetS components in each quartile category, as β regression and 95% CI, are presented in Table 4. Dietary TAC were negatively associated with WC, FPG, TG and BP, and positively associated with HDL-c levels; these associations strengthened across increasing dietary TAC.

**Table 4:** The association of dietary TAC and metabolic syndrome components at baseline (2006–2008): Tehran Lipid and Glucose Study¹

	**( *n* = 1938)**				
	** *Q1* **	** *Q2* **	** *Q3* **	** *Q4* **	** *P* for trend**
Total antioxidant capacity					
*Range*	< 16788	16788-22518	22518-29249	> 29249	
*Mean*	15333	20842	24648	29857	
Waist circumference	0	-1.6 (-2.56, -0.72)	-2.22 (-3.14, -1.29)	-3.89 (-4.82, -2.95)	*<0.01*
Fasting blood glucose	0	-2.41 (-5.22, 0.40)	-2.38 (-5.19, 0.44)	-3.02 (-5.87, -0.17)	*<0.01*
Serum triglycerides	0	-9.27 (-19.70, 1.17)	-10.54 (-21.0, -0.08)	-19.22 (-29.8, -8.64)	*0.24*
HDL-cholesterol	0	0.28 (-0.96, 1.53)	1.31 (0.06, 2.57)	2.24 (0.97, 3.5)	*<0.01*
Systolic blood pressure	0	-0.17 (-1.74, 1.48)	-1.52 (-3.21, 0.39)	-2.53 (-4.41, -0.65)	*<0.01*
Diastolic blood pressure	0	-0.01(-1.15, 1.34)	-1.23 (-2.45, -0.19)	-2.19 (-3.33, -0.97)	*<0.01*

^1^Data are β regression and 95 % CI (Linear regression models were used with adjustment for age, sex, body mass index, physical activity, smoking status, energy and macronutrient intakes; Dietary potassium intake (mg/1000 kcal/d) was additionally adjusted for systolic and diastolic blood pressure).

**Figure 1:**
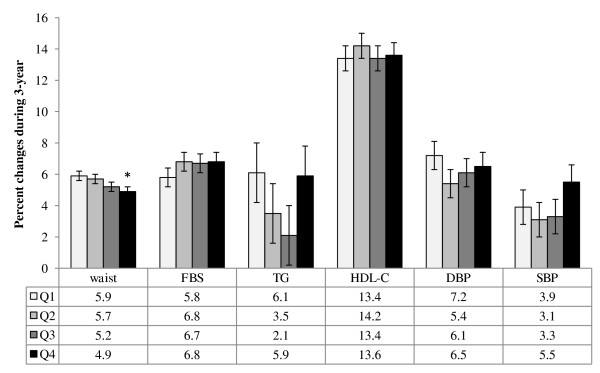
**Percent change of the MetS components across quartile categories of dietary TAC during 3-year follow-up.** General linear models with adjustment for potential confounding variables were used to compare the percent changes of the MetS components between the quartile categories of the dietary TAC (^*^P < 0.05). FBS: Fasting blood glucose, TG: Triglycerides, HDL-c: High density lipoprotein cholesterol, SBP: Systolic blood pressure, DBP: Diastolic blood pressure.

**Figure 2:**
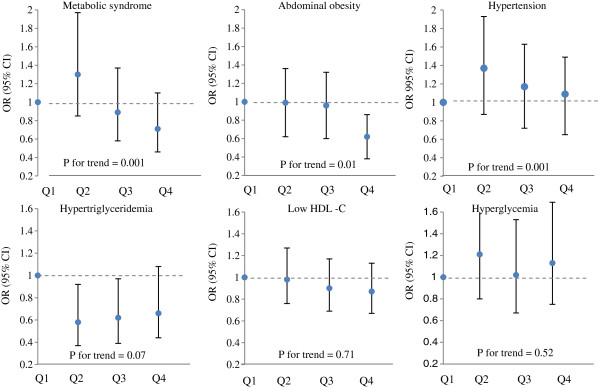
**Odds and 95 % confidence interval for occurrence of the MetS and its components in each quartile categories of dietary TAC after 3-year follow-up.** Logistic regression models with adjustment for potential confounding variables were used to OR estimation.

The mean percent of 3-year changes in MetS components are provided in Figure 1. Participants with the highest TAC score had lower abdominal fat gain during the study follow-up. The odds ratio and 95% CI for the MetS and its components across dietary TAC quartiles are shown in Figure 2. After adjustment for potential confounding variables, the chance of having the MetS, abdominal obesity and hypertension after a 3 year follow-up decreased across the increasing dietary TAC quartile (*P* for trend < 0.01). Dietary TAC over 1080 μmolTE/100 g of food, resulted in 38% decrease the risk of abdominal obesity (OR = 0.62, 95% CI = 0.38-0.99).

## Discussion

The most important findings of this study were an inverse association between dietary TAC and the MetS components at the baseline examination, and the lower occurrence of abdominal obesity after a 3-year follow-up in individuals who had higher dietary TAC. Higher intake of dietary antioxidants also resulted in lower weight and abdominal fat gain during the study period. However although there was no significant decrease in the occurrence of MetS and other metabolic disorders, a significant decreasing trend in the chance of having the MetS and hypertension along with the increase of dietary TAC was observed.

The association of dietary TAC and the MetS components at baseline observed in our study are in agreement with other similar studies that indicated dietary total antioxidant intakes were negatively correlated with lower fasting blood glucose, insulin concentrations, and homeostasis model assessment of insulin resistance in healthy subjects as well as pre diabetic and diabetic patients [16]. Dietary TAC also had inverse association with abdominal obesity, glycemia, total cholesterol to HDL-c ratio, TG, oxidized LDL-C concentration [13] and hs-CRP concentration [15]. Higher intakes of total antioxidant in another cross-sectional study were negatively related with body mass index, systolic blood pressure, serum glucose and free fatty acids [24]. Plasma TAC as a relevant representative indicator of dietary TAC also had a consistent and independent relationship with all obesity indices in men and women [25].

Similar to data available in the current study higher dietary TAC was introduced better diet quality and was related to higher consumption of fruits and vegetables, whole grains, legumes and nuts. The favorable effects of these antioxidant rich foods on improvement of lipid profiles, glucose homeostasis, ameliorating insulin resistance, adiposity and obesity have been investigated in pre-clinical and some clinical studies. Current data suggest that potential effects of food antioxidants such as polyphenols, carotenoids and vitamins may occur through modification of lipids and carbohydrate metabolism, increased insulin sensitivity, and regulation of both appetite and adipocytokines [26]. The anti-obesity effects of high TAC diet during 3-years, investigated in our study may be helpful in validating the oxidative stress-induced obesity hypothesis [27]. Based on this hypothesis, a potential therapeutic role has been considered for dietary antioxidant supplementation in reduction of body weight or improvement of several obesity related disorders [28,29]. In addition, dietary antioxidants also affect other aspects of obesity-related metabolic pathways including inhibition of intestinal fat abortion, promotion of catabolism in adipose tissue, inhibition of proliferation, differentiation, angiogenesis in pre adipocytes, and induction of apoptosis in mature adipocytes [30,31]. Some other dietary antioxidant could prevent adiposity by regulation of brown adipose tissue metabolism and increase thermogenesis, decrease adiponectin and leptin gene expression in adipocytes [32,33].

Since, to our knowledge, the longitudinal association between dietary TAC and MetS components has not yet been evaluated, it is not possible to compare and discuss our longitudinal findings with those of others; hence inevitably we discuss here the effects of serum antioxidant and antioxidant supplementation on the risk of MetS and its components. In a 7.5 yr follow-up study, baseline serum antioxidant concentration including, serum β-carotene and vitamin C was negatively, while plasma zinc was positively associated with incidence risk of the MetS; serum concentrations of vitamin E had no influence on occurrence of the MetS [7]. Clinical trials with antioxidants supplementation showed inconsistent results regarding their ability to reduce the risk of MetS and other related metabolic disorders. No beneficial effects on fasting blood glucose, and the risk of hypertension and MetS, and adverse effects on lipid profiles were observed in longitudinal supplementations with nutritional doses of antioxidant vitamins and minerals [7,8]. Considering the possible different effects of dietary sources or supplement sources of antioxidants, longitudinal studies are needed to investigate the impacts of high TAC diets on several aspects of human health.

Some limitations of the current study should be considered; usual dietary intakes of participants were only assessed at baseline, while several evaluations of dietary intakes could have increased the validity of the results. Using the USDA FCT rather than a complete Iranian FCT is another limitation.

## Conclusion

In conclusion, we demonstrated that higher dietary antioxidant intakes have favorable effects on metabolic disorders and more interestingly prevent subsequent weight and abdominal fat gain during a 3-year follow-up. This study provides further evidence to recommend antioxidant-rich foods as a useful tool in health promotion and disease prevention. In addition to current guidelines recommending increase in consumption of whole-plant foods, focusing on food selection based on antioxidant content of foods certainly would definitely be more effective.

## Competing interest

The authors declare that they have no conflict of interest.

## Authors' contribution

The project was designed and implemented by Z.B and P.M. Data were analyzed and interpreted Z.B and M.G. Z.B, M.G, N.SH and F.A prepared the manuscript. P.M, and F.A supervised overall project. “All authors read and approved the final version of manuscript”.
